# The Actin-Binding Protein Capulet Genetically Interacts with the Microtubule Motor Kinesin to Maintain Neuronal Dendrite Homeostasis

**DOI:** 10.1371/journal.pone.0003054

**Published:** 2008-08-25

**Authors:** Paul M. B. Medina, Ryan J. Worthen, Lawrence J. Forsberg, Jay E. Brenman

**Affiliations:** 1 Neuroscience Center, UNC Chapel Hill School of Medicine, Chapel Hill, North Carolina, United States of America; 2 Department of Cell and Developmental Biology, UNC Chapel Hill School of Medicine, Chapel Hill, North Carolina, United States of America; Centre de Regulacio Genomica, Spain

## Abstract

**Background:**

Neurons require precise cytoskeletal regulation within neurites, containing microtubule tracks for cargo transport in axons and dendrites or within synapses containing organized actin. Due to the unique architecture and specialized function of neurons, neurons are particularly susceptible to perturbation of the cytoskeleton. Numerous actin-binding proteins help maintain proper cytoskeletal regulation.

**Methodology/Principal Findings:**

From a *Drosophila* forward genetic screen, we identified a mutation in *capulet*-encoding a conserved actin-binding protein-that causes abnormal aggregates of actin within dendrites. Through interaction studies, we demonstrate that simultaneous genetic inactivation of *capulet* and *kinesin heavy chain*, a microtubule motor protein, produces elongate cofilin-actin rods within dendrites but not axons. These rods resemble actin-rich structures induced in both mammalian neurodegenerative and *Drosophila* Alzheimer's models, but have not previously been identified by loss of function mutations *in vivo*. We further demonstrate that mitochondria, which are transported by Kinesin, have impaired distribution along dendrites in a *capulet* mutant. While Capulet and Cofilin may biochemically cooperate in certain circumstances, in neuronal dendrites they genetically antagonize each other.

**Conclusions/Significance:**

The present study is the first molecularly defined loss of function demonstration of actin-cofilin rods *in vivo*. This study suggests that simultaneous, seemingly minor perturbations in neuronal dendrites can synergize producing severe abnormalities affecting actin, microtubules and mitochondria/energy availability in dendrites. Additionally, as >90% of Alzheimer's and Parkinson's cases are sporadic this study suggests mechanisms by which multiple mutations together may contribute to neurodegeneration instead of reliance on single mutations to produce disease.

## Introduction

Actin is a major component of the cellular cytoskeleton and one of the most abundant cellular proteins. However, actin must be exquisitely regulated during cell migration, cell adhesion, cell division, and many other essential cellular functions. Actin also forms the core of many cellular structures including filopodia, lamellipodia, microvilli and stress fibers. Actin exists predominantly in one of two forms: monomeric actin (G-actin) and filamentous actin (F-actin). The inter-conversion between these two actin forms is tightly regulated by a diverse array of proteins that bind actin directly or indirectly. Actin depolymerizing factor (ADF), also known as Cofilin, represents one actin-binding protein that can disassemble actin by severing and depolymerizing actin filaments (reviewed by [1], [2], [3], [4]) However, actin severing can also contribute to actin assembly by generating actin fragments with exposed ends to seed new actin polymerization [5].

Dephosphorylation of Cofilin increases its activity, and Cofilin can bind both G- and F-actin (reviewed by [6]). Cofilin binding to F-actin, however, changes the filament conformation eliminating the phalloidin-binding site making the filaments phalloidin negative [7]. In addition, high levels of actin-bound Cofilin can also self assemble into aggregates [8], [9], [10], [11]. The totality of experiments suggests that at any given moment, net actin polymerization and status depends not simply on actin levels, but on the balance of many distinct actin-regulatory factors and their activation status.

Another actin-binding protein that works in conjunction with Cofilin is Cyclase-Associated Protein (CAP), which exists in all eukaryotes [12]. CAP (also known as SRV2 in yeast) recycles Cofilin during actin depolymerization by displacing Cofilin from Cofilin-bound G-actin, thereby freeing Cofilin for another round of actin binding and depolymerization [13], [14]. Conversely, CAP may also function during actin polymerization by transferring G-actin to Profilin, which in turn adds the G-actin to the growing end of F-actin [15], [16], [17]. Previously, Capulet (Capt), the *Drosophila* CAP orthologue, has been shown to be involved in a number of developmental processes including oocyte polarity [18], cell morphogenesis [19], bristle morphogenesis [20], compound eye development [20] and wing disc dorsal/ventral pattern formation [19]. Capulet also has specific roles in neuronal function in *Drosophila* as it works directly with Abl to control axon guidance [21].

In neurons, extremely long neurites filled with G-actin must regulate the formation of F-actin in response to dynamic events such as synapse formation or axon guidance during sensation of chemo-attractive/chemo-repulsive cues. In addition, formation of ectopic F-actin must be suppressed to avoid physical blockages that could impede important transport functions within relatively thin neurites and produce deleterious cellular effects. For example, neurites contain a microtubule-rich cytoskeleton that provides a physical scaffold for delivery-both in anterograde and retrograde directions-for cargoes required to maintain proper neuronal function. Energy-dependent molecular motors, including dyneins and kinesins are ATPases that physically help deliver targeted cargoes by directional movement along these microtubules. In particular the kinesin superfamily protein KIF5 can transport diverse cargoes including membranous organelles, cytoskeletal proteins, and mRNAs (reviewed by [22]).

Forward genetic screens in model organisms provide one potential method for identifying molecules that regulate actin dynamics *in vivo*. *Drosophila* forward genetics has been applied to identify molecules required for processes ranging from embryo patterning to axon guidance. From a previous genetic screen, we identified a particular mutation that affected the accumulation of an actin, GFP fusion protein (actin::GFP), intriguingly only within neuronal dendrites but not axons [23]. We demonstrate here that loss of function mutations in *capulet*, are responsible for numerous actin-related phenotypes. Further, loss of function mutations in different genes synergize to produce severe phenotypes not observed by single loss of function mutations. In particular, we show that *capulet* genetically interacts with *kinesin heavy chain* and affects kinesin-mediated transport in the dendrites including mitochondrial distribution. Remarkably, the double mutant *capulet* and *khc* phenotype includes the formation of actin rods similar to those described in a *Drosophila* Alzheimer's model and in mammalian tissue culture neurodegeneration models. This study suggests a mechanism by which robust phenotypes may result from multiple seemingly mild impairments that interact with each other.

## Results

### Identification of the capulet phenotype in dendrites

We previously conducted a forward genetic screen to isolate mutations affecting neuronal dendrite development [23]. One particular mutant, previously named “*punctate*”, displayed actin::GFP-rich accumulations within dendrites but not within axonal shafts. However, neuron numbers and gross aspects of neural development including dendritic filopodia formation and dendritic branching appeared relatively normal within this mutant. These actin::GFP puncta, which are absent in wild type larvae, occur at an average of 38 puncta (standard deviation = 10) in each sensory neuron dorsal cluster of the *punctate* mutant. To determine the molecular lesion responsible for this phenotype, we used deficiency mapping and identified two molecularly defined deficiencies, *Df(2L)Exel7006* and *Df(2l)Exel6004*, that failed to rescue the lethality of the *punctate* mutant. These two deficiencies overlapped by only 36 nucleotides within the *Drosophila capulet* (*capt*) gene. The Capulet protein encodes 424 amino acids with 3 recognizable motifs, namely a polyproline domain, a WH2 domain and two CARP domains. To confirm that *capt* was the affected gene, we sequenced the *capt* locus from the *punctate* mutant and found a nonsense mutation that changed amino acid lysine 304 to a premature stop codon. This mutant allele of *capt*, termed *capt* ^K304^, eliminated the last 121 amino acids of the Capulet protein, which includes both CARP domains. CARP domains are secondary structure *predicted* homology, not amino acid similarity, found in both Capulet and a gene mutated in Retinitis Pigmentosa [24]. The CARP domains bind to monomeric actin, while the polyproline stretch and WH2 domain also mediate interaction with the actin cytoskeleton [25], [26], [27], [28].

**Figure 1:**
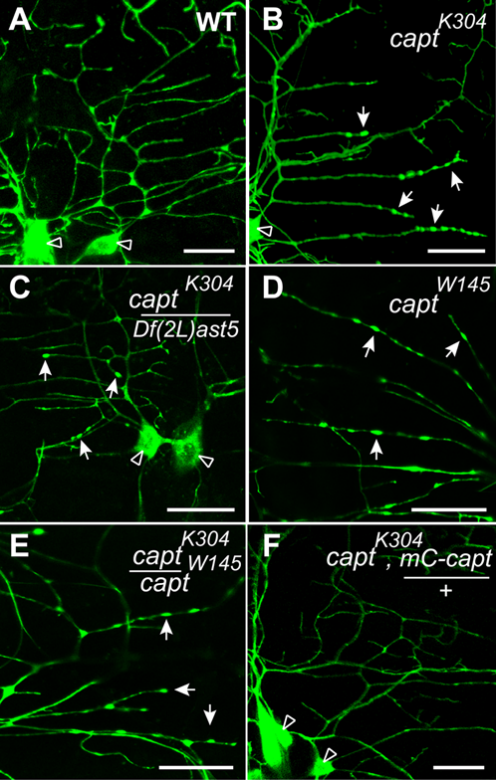
The *capulet* (*capt*) mutant demonstrates abnormal actin::GFP accumulation in neuronal dendrites. PNS neuronal dendrites in a live wild type (WT) larva (A) show dispersed labeling of actin::GFP in cell bodies (open arrow) and dendrites, while *capt* ^K304^ mutant dendrites display actin::GFP punctate-like accumulates within dendritic shafts (B, arrows). (C) A chromosomal deletion of *capt* produces a similar phenotype to the point mutant identified from the screen when transheterozygous. An independent allele of *capt* (*capt* ^W145^, D) that fails to complement *capt* ^K304^ lethality, produces a similar dendrite phenotype when transheterozygous (E). (F) The *capt* ^K304^ phenotype is rescued by a transgene encoding the *mCherry::capulet* (*mC-capt*) fusion protein. Arrows indicate abnormal actin::GFP accumulation in dendrites. Scale bar is 20 µm. (All larvae contain *Gal4 109(2)80*, *UAS-actin::GFP* (green signal) for visualization unless otherwise noted; in all figures the dorsal cluster of sensory neurons are shown with anterior toward the left and dorsal toward the top.)

To confirm that the dendrite actin::GFP phenotype was caused by mutations in *capt*, we determined whether deficiencies that uncover the *capt* locus would also display the actin::GFP phenotype seen in *capt* ^K304^ (Figure 1B). We successfully recombined the *actin::GFP* transgene onto *Df(2L)ast5*, a deficiency that lacks the *capt* locus, which also fails to complement the lethality of *capt* ^K304^. Indeed, we observed the actin::GFP phenotype when the deficiency was transheterozygous with *capt* ^K304^ (Figure 1C). We subsequently obtained a previously published allele of *capt* that encoded a nonsense mutation at tryptophan 145 (*capt* ^W145^), and introduced the *actin::GFP* transgene into this mutant background. The *capt* ^W145^ mutant also displayed the same actin::GFP phenotype when either homozygous or transheterozygous with *capt* ^K304^ (Figure 1D–E). Similar phenotypes were observed with two additional P element insertion alleles *capt* ^k01217^ and *capt* ^06955^ (data not shown). In addition to demonstrating nearly identical phenotypes with different alleles, we tested whether expression of a *capt* transgene in a *capt* ^K304^ background would be sufficient to rescue the actin::GFP phenotype. To accomplish this, we made a fluorescently-tagged *capt* construct (*UAS-mCherry::capulet*) and recombined it onto the *capt^K304^* chromosome. The *capt^K304^*, *UAS-mCherry::capulet* recombinant did not display any actin phenotype (Figure 1F), thus confirming the phenotype is due to the *capulet* mutation.

### Capulet function in neurons

**Figure 2:**
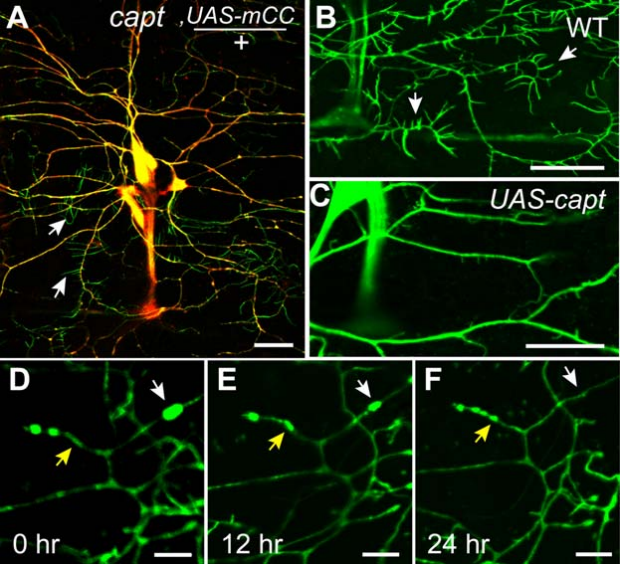
Capulet modulates actin structure in neuronal dendrites. (A) PNS neurons expressing a rescuing transgene of mCherry::capulet (mCC, red) demonstrate co-localization within the soma, dendrites and axons (yellow) with actin::GFP but not within dendritic filopodia (green, arrows), which are enriched for F-actin. (B) Wild type PNS neuronal dendrites with abundant filopodia (arrows). (C) In contrast, overexpression of Capulet (*UAS-capt*), a monomeric actin-bindng protein, reduces the number of dendritic filopodia (arrows). (D–F) Time lapse analysis of the identical *capt* ^K304^ dendritic segment expressing actin::GFP demostrates that actin::GFP accumulations are dynamic in the *capulet* mutant. White arrows indicate disappearing actin::GFP accumulates while yellow arrows indicate the simultaneous appearance of actin::GFP accumulations within the same dendrite. Scale bar is 20 µm in A–C and 5 µm in D–F. (All larvae contain *Gal4 109(2)80* and *UAS-actin::GFP* (green) and/or UAS-mCC (red) for visualization.)

In yeast and *Drosophila* epithelial cells, capulet phenotypes are most obvious at cortical actin patches [29] and adherens junctions [30] respectively. Neurons, unlike yeast and epithelial cells, have more structurally isolated compartments due to their complex morphology. To examine the subcellular localization of Capulet in PNS neurons, we performed antibody immunostaining and expressed the *UAS-mCherry::capulet* transgene (*UAS-mCC*). In both cases, Capulet localized within cytoplasmic compartments including axons, cell bodies and dendritic shafts but did not localize to the F-actin-rich dendritic filopodia [31] (Figure 2A). This is consistent with a role for Capulet as an actin monomer-binding protein since filopodia are primarily F-actin structures [32], [33]. Capulet is thought to function by inhibiting inappropriate actin polymerization within cells as demonstrated by mutations in *Drosophila*, whether in epithelial cells [30] or eye cells [20] that demonstrate *increased* F-actin in loss of function *capt* mutant cells. The converse hypothesis suggests that elevated *capt* function might *decrease* F-actin formation. We therefore tested this putative role on an F-actin-rich cellular structure, dendritic filopodia, by overexpressing wild type Capulet. We found that overexpression did suppress formation of F-actin-rich dendritic filopodia (Figure 2C), consistent with a role for Capulet as a potential inhibitor of filamentous actin formation and G-actin monomer-binding protein.

Actin-binding proteins can directly or indirectly influence the balance between free G-actin monomers and polymerized F-actin. We wondered whether the actin::GFP accumulations were stable or transient in nature. We observed that there did not appear to be a substantial net increase in actin::GFP-rich aggregates in older *capt* ^K304^ larvae compared to younger *capt* ^K304^ larvae, suggesting that actin::GFP puncta did not accumulate over time. To directly assess the dynamics of the actin::GFP phenotype we performed time lapse imaging of actin::GFP expressing *capt* mutant larvae. Time lapse analysis revealed that the actin::GFP phenotype is dynamic with accumulations dissipating within dendrites, while simultaneously new accumulations can form within a different part of the same dendrite (Figure 2D–F). This suggests the possibility that Capulet functions to regulate the balance between actin in filaments and actin in soluble monomeric form within dendrites.

### Capulet and cofilin interaction in neurons

**Figure 3:**
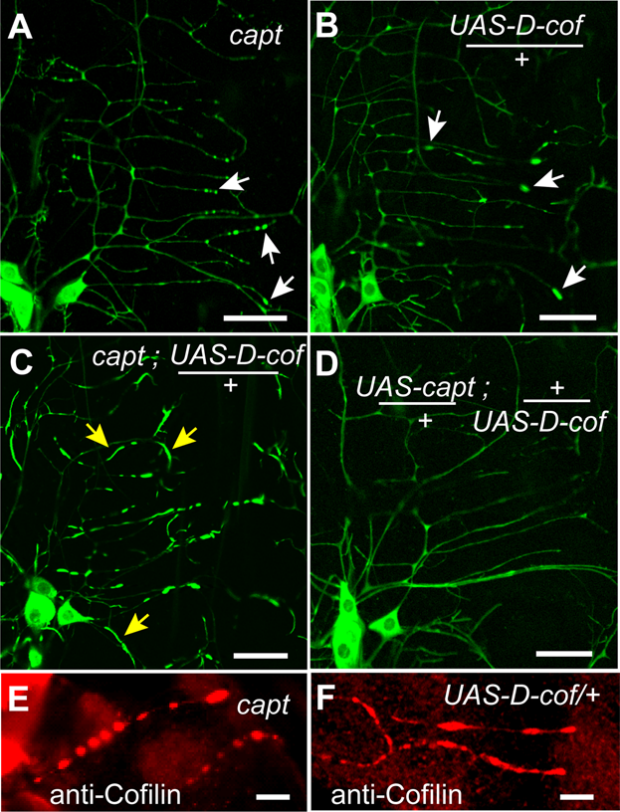
*capulet* and *Drosophila* ** *cofilin* (*D-cof* (or *twinstar*)) genetically antagonize each other in regulating dendritic actin. Neuronal dendrites exhibit similar actin::GFP accumulation phenotypes in either a *capt* loss of function mutant (A, arrows) or upon *cofilin* overexpression (*UAS-D-cof*) (B, arrows). Actin::GFP forms longer rod-like actin::GFP structures when *cofilin* is simultaneously expressed in a loss of function mutant *capt* background (C, yellow arrows). However, dendrites appear normal when *cofilin* and *capulet* are simultaneously co-expressed (D). Actin accumulates in *capt* mutants (E) immunostain for Cofilin (red), as do actin accumulates and longer actin rods that form upon Cofilin overexpression (F, *UAS-D-cof*). Scale bar is 20 µm in A–D and 5 µm in E–F. (All larvae contain *Gal4 109(2)80*, *UAS-actin::GFP* (green) for visualization in addition to any Cofilin immunostaining (red).)

Previous studies demonstrate that Capulet interacts with Cofilin to promote actin turnover and that the absence of Capulet can lead to formation of Cofilin/actin aggregates [34]. We utilized genetic analysis to examine the genetic relationships between *capulet* and *cofilin*. Overexpression of Cofilin in mammalian cells has been demonstrated to lead to the formation of ADF/Cofilin (AC)-actin rods [35]. These investigators proposed that high levels of actin bound Cofilin can lead to self-assembly and actin-rod or actin-puncta formation [35]. Upon over-expression of Cofilin in wild type da neurons we observed formation of actin::GFP accumulations with occasional rod-like structures forming (Figure 3B). However, these actin-rods appear to be greater in number and longer when *cofilin* is overexpressed within a *capt* ^K304^ background (Figure 3C), suggesting that *capulet* helps reduce actin-rod formation by interacting genetically with *cofilin*. Conversely, no actin rods form when *cofilin* and *capt* are simultaneously over-expressed (Figure 3D), suggesting that capulet and cofilin genetically antagonize each other. These results, however, are also consistent with the biochemical models in which Capulet helps recycle Cofilin and enhance it's depolymerization activity [13], [14], [34].

In mammalian cells, AC-actin rods do not usually stain with phalloidin but are instead decorated with Cofilin [36]. We phalloidin stained the *capulet*, *khc* double mutant and immunostained for Cofilin. Both the actin puncta and the actin rods failed to stain with phalloidin yet both contained abundant Cofilin (Figure 3E–F), suggesting they share some features with AC-actin rods described in mammals.

### Capulet mutations alter mitochondria distribution in dendrites

**Figure 4:**
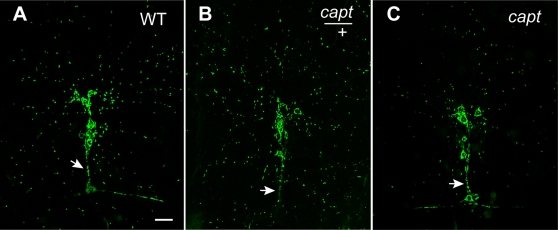
Capulet function is required for normal mitochondrial distribution in neuronal dendrites. In wild type neurons mitochondria (visualized with mito::GFP, green) are most abundant in cell bodies and distributed throughout the axons and dendrites. (B) A *capulet* heterozygote shows a similar mitochondrial distribution. (C) The *capulet* mutant shows normal mitochondrial distribution in cell bodies and axons but decreased distribution in distal dendrites. Arrow indicates the axon bundle. Scale bar is 20 µm. (All larvae contain *Gal4 109(2)80* and *UAS-mito::GFP* (green) for visualization.)

Depletion of ATP in mammalian cells has been shown to generate AC-actin rods. Further, in mammalian neurites the AC-actin rods fill the diameter of neurites essentially blocking transport within the neurite and severely disrupting the microtubule network (reviewed by [37]). Due to the long, thin architecture of sensory dendrites, it is possible that the formation of the Cofilin-decorated actin puncta in the *capt* ^K304^ mutant could also hinder transport within these dendrites. Mitochondria are relatively large organelles that are actively transported along microtubule tracks within dendrites to provide ATP throughout the dendritic tree. We determined whether the transport of mitochondria within dendrites is affected in *capt* ^K304^ by expressing mito-GFP, a mitochondrial targeting sequence fused to GFP, in the PNS to visualize the distribution of mitochondria. In a *capt* ^K304^/+ heterozygote, the mitochondria are dispersed throughout the length of the dendrites, whereas in the *capt* ^K304^ homozygote, there are significantly fewer mitochondria dispersed in the dendrites with approximately a 50% reduction in dendritic numbers (Figure 4A–C). However, the distribution of mitochondria in the cell bodies and in the axons between the *capt* heterozygote and *capt* homozygote are comparable. This suggests that Capulet could help maintain dendrites clear of actin polymerization that may otherwise impede microtubule-based transport such as mitochondria transport.

### Capulet genetically interacts with kinesin heavy chain

**Figure 5:**
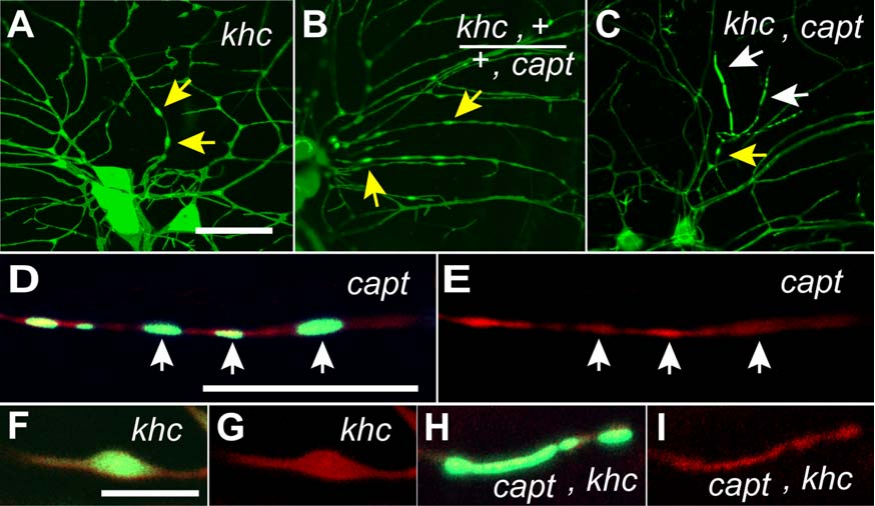
*capulet* genetically interacts with *Kinesin heavy chain* (*khc*) to produce actin rod-like structures in dendrites. (A) The *khc* mutant phenotype demonstrates membrane swellings in dendrites (yellow arrows). (B) A genetic interaction between *khc* and *capt* revealed by the dendrite swelling phenotype (yellow arrows) in a trans-heterozygote not observed in either single heterozygote. (C) The simultaneous decrease of *khc* and *capt* function produces elongate actin rod-like structures in dendrites (white arrows) and smaller swellings (yellow arrow). The *capulet* actin puncta (white arrows-D, E) and the *capulet/khc* double mutant actin rods (H, I) do not produce membrane swellings like that observed with the single *khc* mutant (F, G). (The plasma membrane was visualized with myristoylated monomeric Red Fluorescent protein (red, D–I)). Yellow arrows indicate swellings, while white arrows indicate actin rods (C) or actin puncta (D, E). Scale bars are 20 µm in A and D, 5 µm in F. (All larvae contain *Gal4 109(2)80* and *UAS-actin::GFP* (green) or *UAS-myristoylated RFP* (red, membranes) for visualization.)

We wondered what genes, other than actin-binding proteins, might be relevant to *capulet* function, which could also explain the mitochondrial phenotype. Previous studies in *Drosophila* have demonstrated that mutations in *capulet* and *kinesin heavy chain* (khc) had similar phenotypes in establishing anterior-posterior asymmetry. The anterior localization of *bicoid* mRNA and posterior localization of *oskar* mRNA are essential in organizing the anterior-posterior asymmetry in *Drosophila* oocytes. One study documented that a mutation in *capt* alters the posterior localization of *oskar* mRNA [18]. In a separate study, a mutation in *kinesin heavy chain* (*khc*) was also shown to perturb *oskar* mRNA distribution [38]. In addition, the major mediator of transport of mitochondria in axons and dendrites is Kinesin heavy chain (Khc)[39]. Since both *capt* and *khc* mutations produced the same *oskar* mRNA mislocalization phenotypes in oocytes and Khc transports mitochondria, we investigated whether there was any genetic interaction between *capt* and *khc*. To determine whether genetic interactions exist between *capt* and *khc*, we placed an *actin::GFP* transgene in a *khc* mutant (*khc* ^k13314^) background. Homozygous *khc* ^k13314^ mutants exhibited swellings in dendrites (Figure 5A), similar to the axonal swellings previously observed in *khc* mutants [40], whereas these swellings were absent in a *khc* ^k13314^ heterozygote. When we crossed the *capt* ^K304^ line with the *khc* ^k13314^ line, the *capt*, *khc* transheterozygote showed dendritic swellings, confirming that these two genes genetically interact in a doubly heterozygous animal (Figure 5B). In a *capt*, *khc* double loss of function mutant, both the actin::GFP puncta and dendritic swellings, seen in *capt* and *khc* single mutants respectively, occurred at higher frequency (Figure 5C), suggesting that they enhance each other. Strikingly, however, we also observed the formation of elongate actin rods in the double mutant (Figure 5C), not seen in either single mutant. Actin::GFP puncta in *capt* mutants had an average length of 1.23 µm (standard deviation = 0.21 µm), whereas the actin::GFP rods we observed in the *capt*, *khc* double mutant had an average length of 5.97 µm (standard deviation = 1.75 µm). The average number of actin rods (>2 µm in length) found in the dorsal cluster sensory da neurons was ∼7 per cluster (mean = 7.33, standard deviation = 2.77). Importantly, actin rods were never seen in wild type larvae. We tested the genetic interactions between *capt* and *khc* using a different allele of *capt* (*capt* ^W145^) or a different allele of *khc* (*khc* ^e02141^) and we were able to replicate the same findings (data not shown).

Alteration of kinesin function is associated with swellings in the plasma membrane in axons [40], [41], [42]}. We visualized the dendrite membrane by expressing myristoylated-mRFP (red fluorescent protein), which targets to the plasma membrane, to determine whether or not the actin::GFP accumulations affect the diameter of the surrounding dendrite. Surprisingly, unlike kinesin mutations that are associated with membrane swellings, neither the actin puncta nor the actin rod-like structures are associated with localized swellings. (Figure 5D–I).

**Figure 6:**
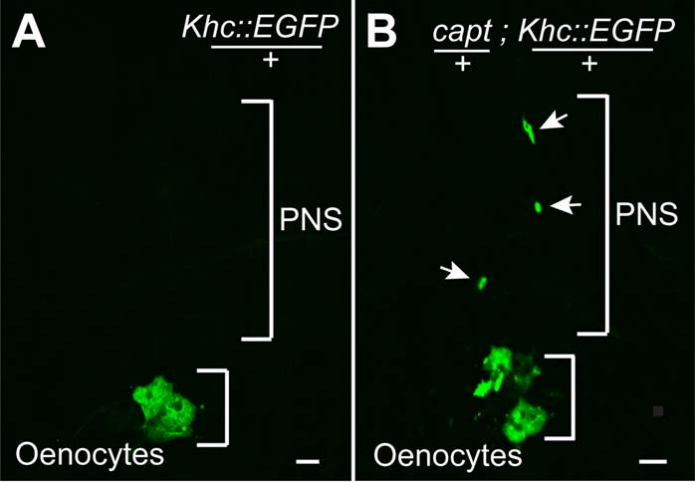
Reduced *capulet* function leads to Kinesin aggregation in neuronal cell bodies. Kinesin heavy chain tagged with GFP (*Khc::EGFP*) expressed in a heterozygous *capulet* mutant background (capt/+) demonstrates aggregation in dorsal cluster sensory neuron cell bodies (arrows, B) but not in a wild type (for *capulet*) animal (A). Possibly, this phenotype is due to impaired transport of Khc::EGFP out of cell bodies (white arrows, B) and into dendrites. Note abundant Khc::EGFP expression in oenocytes in the same field to control for Khc::EGFP expression levels in other cell types. (All larvae contain *Gal4 109(2)80, UAS-Khc::EGFP* for visualization.)

To compare the neuronal distribution of Khc in a WT and *capt* background, we expressed a *Khc::EGFP* transgene in the PNS neurons. We could not easily visualize Khc::GFP along the dendrites of PNS neurons in both WT and *capt* backgrounds with a single overexpressing transgene. However, Khc::EGFP appears to accumulate in the cell bodies in a *capt* heterozygote which is not observed in a WT background (Figure 6 A–B), suggesting that Khc::EGFP movement from cell body to dendrites is disrupted when Capt levels are reduced to half. We attempted to make a recombinant to express more Khc::GFP to more easily visualize dendrites, however, we discovered that the *capt*, *Gal4 109(2)80*; *UAS-Khc::GFP* transheterozygote is synthetic lethal and dies at the pupal stage. This observation further supports a *capt* and *khc* genetic interaction. Possibly, the increased amount of Khc::GFP exceeds the level of Capt function in a heterozygote required for unimpeded Kinesin function.

## Discussion

Regulation of actin dynamics has important consequences in all cell types but special significance in neurons as dendritic spines – the sites of synapse formation in the brain – and axon growth cones are highly enriched for F-actin. In addition, neurites, either axons or dendrites, span very long distances from the cell body and therefore must allow continued transport of proteins to and from the cell body to either axon termini or dendritic spines. Blockage of relatively small diameter dendrites could hinder this transport and presumably have deleterious affects on neuronal function.

Capulet plays a vital role in regulating actin dynamics and can reduce filament formation by binding/sequestering actin monomers and preventing new polymerization or by recycling Cofilin activity by removing actin monomers from it, thus freeing Cofilin for another round of actin severing and depolymerization [43]. Consistent with this function, multiple screens in *Drosophila* have identified *capulet* loss of function mutations that lead to ectopic increased F-actin accumulation [20], [30]. When Capulet function is reduced, endogenous Cofilin may have less F-actin depolymerizing activity leading to actin filament accumulation. Alternatively, very high levels of Cofilin, generated by over-expression experiments in mammalian cells or in this study with transgenic animals, can exceed the capacity of Capulet to recycle Cofilin depolymerizing activity, leading to self-assembly of actin-bound Cofilin into AC rods. In essence, the balance between Capulet and Cofilin levels and their activity determines the net actin polymerization status. Our genetic studies support various biochemical and tissue culture models whereby decreases in *capulet* function lead to increased actin in small aggregates, while over-expressed Cofilin in a *capulet* mutant background leads to larger accumulation in actin rod-like structures.

### Alterations in actin can affect microtubule-based function

Perturbations in Capulet function not only affect actin dynamics, but also microtubule-based processes. A Recent study demonstrated that the actin nucleators, Spire and Cappuccino, have major affects on the regulation of the microtubule network and cytoplasmic flows in the *Drosophila* oocyte [44]. Cappuccino and Spire are required for the formation of an actin meshwork that suppresses Kinesin motility along microtubules to help maintain proper anterior-posterior polarity in the oocyte. In this example, regulation of the actin meshwork affects microtubule-based Kinesin function.

**Figure 7:**
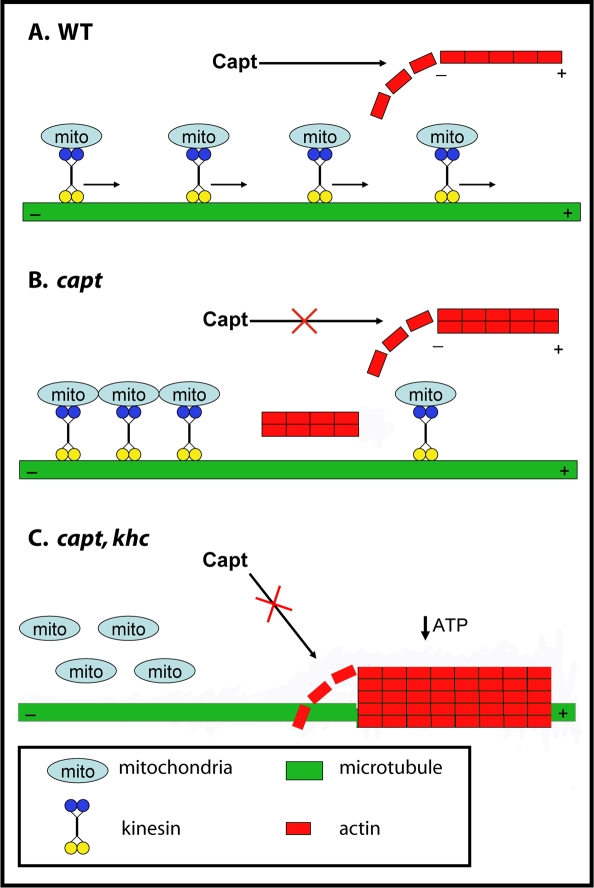
Model suggesting that Kinesin Heavy Chain and Capulet may cooperate to maintain dendrite homeostasis. (A) Capulet normally inhibits excessive/ectopic F-actin polymerization and thus allows free movement of motor proteins, including Kinesin, along microtubules in dendrite shafts. Kinesin cargoes such as mitochondria can be freely distributed along dendrite shafts. (B) Elimination of Capulet activity leads to ectopic actin aggregates in dendrite shafts that impede microtubule-based transport, but may have less impact in larger diameter axons. (C) When Capulet and Kinesin function are both impaired large actin rods result, possibly from simultaneous decreased mitochondrial transport (and lower ATP production in distal dendrites) and decreased actin filament depolymerization due to lack of Capulet activity.

In our study, Capulet maintains dendrites clear of ectopic actin filaments that otherwise could physically disrupt microtubule-based function, including Kinesin-mediated transport. In addition to other cargoes, Kinesin also transports mitochondria in the anterograde (plus-end) direction along microtubules [38], [45]. Impaired Kinesin function could then diminish mitochondrial transport, which are transported hundreds of microns from the cell body in dendrites, leading to less available ATP. The transheterozygous genetic interaction we observe between *capulet* and *khc* as well as the robust double mutant phenotype that produces actin rods in dendrites suggests a strong cellular interdependence between these two molecules (see model Figure 7).

A fundamental question relates to why we only observe dendrite and not axon phenotypes. It is highly unlikely that *capulet* only functions in dendrites, as roles for *capulet* in actin regulation have already been demonstrated in diverse cell types from follicle epithelial cells to the adult compound eye [20], [30]. The simplest interpretation is that the abundance of actin-based structures in these dendrites, dendritic filopodia, which are not found on axons, represents a larger pool of actin to be regulated. Alternatively, the internal diameters of these dendrites are much smaller than their axons suggesting any impairment of cytoplasmic flow – by diffusion or transport-would be preferentially obstructed in the dendrites. Indeed, the distribution of mitochondria in cell bodies and axons are normal in *capulet* mutants, however, relatively smaller *Drosophila* sensory dendrites would be more susceptible to impaired transport and decreased ATP.

### The role of actin in neurodegenerative disease

The role of altered actin polymerization and aggregation in neurodegenerative disease is relatively unexplored, although some studies have begun to examine actin aggregates in neurodegenerative disease. In Alzheimer's disease brain, aggregates of ADF/Cofilin have been described in association with both amyloid deposits and neurofibrillary tangles [36], [46]}. There are also aggregates of ADF/Cofilin in actin-crystalline structures termed Hirano bodies, which have been documented in distinct neurodegenerative diseases including Alzheimer's and Parkinsons [47]. One might speculate that alterations in actin dynamics may not be a causative factor in neurodegenerative conditions but perhaps a contributing factor to neuronal dysfunction.

Recently, investigators have described an Alzheimer's disease model developed in the *Drosophila* brain with altered actin dynamics. In this study, the authors documented that ectopic expression of a mutant human Tau protein, associated with fronto-temporal dementia, increases the levels of F-actin in the brain [48]}. Further, they observed actin rod structures in the brains of both the *Drosophila* model and in a mouse transgenic tauopathy model. Some of the rods stained for actin, Cofilin and Tau, which are all characteristic of Hirano bodies. It is interesting to note that in their study, co-expression of Cofilin with the mutant Tau protein significantly reduced the effects of expressing mutant Tau alone, suggesting that they genetically antagonize each other. Conversely, over-expression of human wild type Tau enhances the toxicity of β-amyloid (Aβ). These authors suggest that interactions between the microtubule binding protein Tau and actin may be a key mediator of Tau-related neurotoxicity [48]. We did not see a genetic interaction in a heterozygous *capulet* background when expressing the human mutant Tau protein [48] (data not shown). Our results suggest mechanisms by which loss of *capulet* function interacts with impaired Kinesin function to produce similar actin rods. Interestingly, both studies suggest that proteins normally associated with microtubules can genetically interact with the actin cytoskeleton under pathological conditions.

In many neurodegenerative models impaired Kinesin function and the failure of motor-driven transport in long axonal compartments is hypothesized to play a major role in neurodegeneration. This transport failure leads to formation of axonal swellings, which are the physical manifestation of accumulation of axonal cargoes. For example, overexpression of *Drosophila* βAPP results in axonal transport defects, which are enhanced by genetic reductions in the amount of functional Kinesin [49]. βAPP and its proteolytic machinery undergo Kinesin 1-mediated fast anterograde axonal transport in neurons [50]. Interestingly, mutations in Tau, a microtubule binding protein, are also extensively documented in Alzheimer's (reviewed in [51], [52]).

### Are actin rods protective?

Although actin rods are induced under conditions that diminish neuronal function or with expression of neurodegeneration-associated proteins, at least one study postulates that actin rod formation may be a neuroprotective response [35]}. Neuronal cell culture models designed to mimic neurodegenerative insults, including excitotoxicity and oxidative stress, induce formation of ADF/Cofilin-actin rods within neurites. When examined by electron micrographs these ADF/Cofilin-actin rods physically displace microtubules from the neurite by filling it [36]. While these rods would be clearly detrimental to neural function in the short term, in the long term the authors speculate that by possibly inhibiting actin treadmilling the rods may decrease ATP consumption. As an ATPase, active actin treadmilling consumes ATP, which could be problematic under cellular stress. Both the small actin aggregates documented here (by time-lapse analysis), and the actin rods documented in mammalian cells can be dynamic and dissipate and return to normal actin pools, allowing for the possibility that they could be transiently neuroprotective. If the actin rods were neuroprotective they would have to be relatively short-lived. In our investigations, *capt/khc* double mutant larvae do not survive long enough under time lapse to observe the disappearance of actin-rods (data not shown).

In addition to forming actin rods by overexpressing Cofilin or microinjecting Cofilin, rod formation can also be induced simply by depleting ATP. Decreasing ATP would alter numerous cellular pathways. However, Cofilin has a higher affinity for ADP/actin than ATP/actin and by self-assembling ADP/actin/Cofilin into rods, the exchangeable ADP/actin could be removed from the dynamic actin pool decreasing ATP turnover. Interestingly, actin rods are not unique to neurons, as non-neurons under stress can also be induced to form Cofilin positive, phalloidin negative actin rods [53]. Reorganization of actin stress fibers (that stain with phalloidin but not Cofilin) into cytoplasmic and intranuclear actin rods decorated with Cofilin but not phalloidin have also been produced in mouse fibroblasts by exposing the cells to stressors [53].

### Other actin regulators and kinesin function

A second, line of thought suggests that other actin regulatory proteins may directly interact with kinesin and could provide another route for Capulet ultimately affecting Kinesin-mediated transport. Enabled (Ena) is an actin-polymerizing factor that genetically antagonizes the Abl tyrosine kinase (reviewed by [54]). Reduced Ena function has been demonstrated to rescue *khc* phenotypes-paralytic tail flipping and axonal swellings – in a sensitized *Abl* background [55]. These investigators suggest that Ena normally inhibits kinesin-mediated transport by a direct physical association. However, as Ena facilitates actin polymerization, an alternative interpretation could be that unchecked by Abl, excessive Ena activity could lead to ectopic actin polymerization, which could hinder kinesin-mediated transport. Abl, which inhibits Ena activity, has a SH3 domain that could interact directly with Capulet's polyproline domain and Abl and Capulet have already been demonstrated to co-immunoprecipitate [21]. In fact, previously it was demonstrated that Abl and Capulet serve a common function in ultimately promoting repulsion of CNS axons to the midline [21]. It would be consistent for *capulet* and *abl* to also have a common function in promoting kinesin movement in microtubules, as both of these mutants form swellings in neuritic processes when transheterozygous with *khc*. Furthermore, both Abl and Capulet prevent formation of F-actin, either by Capulet depolymerizing F-actin via Cofilin, or by Abl negatively regulating Ena.

In summary, we demonstrate a strong genetic interaction between *kinesin* and *capulet*. A *capulet-khc* transheterozygote forms dendritic swellings, similar to the dendritic swellings seen in a *khc* mutant alone. However, neither reduced function of either *capulet* nor *kinesin* results in actin rods. Only the double mutant of both produces AC-like actin rods observed in numerous animal and cell culture models of neurodegeneration. Taken together, our observations suggest that *capulet* is necessary for kinesin-mediated dendritic transport and in turn cargoes transported by kinesin, like mitochondria, have vital roles in regulating actin dynamics in dendrites. While an appreciation of actin dysregulation as a potential contributor to neurodegenerative disease is just beginning, this study illustrates the severe consequences of altered actin regulation in combination with other defects. In this sense, it is interesting to note that >90% of Alzheimer's and Parkinson's cases are sporadic [36], [56], [57], suggesting that perhaps multiple defects in different proteins/genes may combine to cause sporadic neurodegenerative diseases.

To the best of our knowledge, this is the first case of Cofilin-decorated actin rod formation due to defined genetic loss of function mutations. Previous actin rods have been generated by subjecting cells to stress (ATP depletion, heat shock treatment, oxidative stress by hydrogen peroxide treatment, glutamate-induced excitotoxicity, and DMSO treatment) or by over-expressing proteins such as Cofilin, Tau and β-Amyloid peptide in transgenic models.

## Materials and Methods

### Fly stocks

We follow guidelines from Flybase (www.flybase.org) for nomenclature. Gene names are italicized, whereas proteins are not. To visualize PNS neurons, *Gal4 109(2)80* (Gao et al. 1999) was recombined with *UAS-actin::GFP* (Verkhusha et al. 1999) to produce the *Gal4 109(2)80*, *UAS-actin::GFP* recombinant. The following fly lines were obtained from the Bloomington Stock Center (Bloomington, IN): DrosDel and Exelexis deficiency stocks on chromosome 2, deletion mutants *Df(2L)Exel7006* (stock # 7776), *Df(2L)Exel6004* (stock # 7491) and *Df(2L)ast5* (stock # 6344); *capt* ^E636^ allele which we refer to as *capt* ^W145^ (stock # 5944) and *UAS-capt* line (stock # 5943); *khc* ^k13314^ (stock # 11084) and *khc* ^e02141^ (stock # 18018); *UAS-tsr* (stock # 9235); UAS-myr-mRFP (Stock # 7119), *UAS-mitoGFP* (stock # 8443) and *UAS-Khc::EGFP* (stock # 9647). Second instar larvae were used for all experiments unless otherwise indicated. All flies were maintained at 25°C in yeast-cornmeal (Bloomington recipe) vials and bottles.

### Mapping and gene identification of *punctate*

Virgin females of *punctate* (*punc*/*CyO*) were crossed with males from each stock of the Chromosome 2 Deficiency Kit from Bloomington. We noted which deficiency stocks did not generate F1 progeny with straight wing flies (i.e., *punc*/*def* is lethal). We identified the common chromosomal locus among all the deletions in these stocks, which is within the *capulet* gene. To identify the nature of the EMS mutation, we amplified and sequenced overlapping segments of *capt* from genomic DNA of *punctate* and compared it to the WT *capt* sequence.

### Cloning

To make the *mCherry::capt* (monomeric Cherry::Capulet) fusion construct, *mCherry::Tubulin* was taken out of pcDNA3.1 (gift from the R. Tsien lab) by cutting with *Eco*RI. Both ends of the excised mCherry sequence were made blunt by Klenow fragment (New England BioLabs, Ipswich, MA) treatment and the 3′ end was made sticky by digestion with *Xho*I. The *pUAST* vector was linearized by digestion with *Hind*III and the ends were made blunt by Klenow fragment treatment. The 5′ end of the *pUAST* vector was cut with *Xho*I to create a sticky end. The *mCherry::Tubulin* fragment was then inserted into the *pUAST* vector by blunt-sticky ligation. The *capulet* gene sequence was amplified from the cDNA clone LD24380 by using a forward primer containing a *Bgl*II restriction site and a reverse primer containing an *Xho*I restriction site. The *capulet* PCR product was digested with *Bgl*II and *Xho*I. The tubulin sequence was excised out of the newly constructed *pUAST-mCherry::Tubulin* by digestion with *Bgl*II and *Xho*I and was replaced with the *capulet* sequence. The p*UAS-mCherry::capulet* construct was transformed into electrocompetent DH10B bacteria, amplified and injected into *w* ^1118^ *Drosophila* embryos by P element mediated transformation to make transgenic fly lines (Duke *Drosophila* Transgenic Core).

Polyclonal antibodies against His-Capulet were generated by linearizing the *pET28a* vector with *Bam*H1 and *Xho*I digestion and ligating the amplified *capulet* PCR product (see above) that had been digested with *Bgl*II and *Xho*I. The *capulet* in the *pET28a* vector was transformed into competent cells, amplified, sequence verified, and transformed into BL21 cells. The His-Capulet protein was extracted and purified using the TALON system and sent to QED Bioscience Inc. (San Diego, CA) for antibody production.

### Immunohistochemistry

The dissection of larvae, immunostaining procedures and confocal microscopic visualizations were done essentially as described (Medina et al., 2006). Briefly, the larva was washed with water and placed in a droplet of 4% para-formaldehyde. The head and the tail sections of the larva were excised using a pair of fine scissors and the guts and other visceral organs were pulled out with forceps. An anterior-posterior cut was made along the mid-dorsal area of the larva and the resulting larval fillet was cleaned out of muscle tissue with forceps. The larval fillet was fixed in 4% paraformaldehyde for 20 minutes and then washed with PBT 3 times. This was followed by incubation with a blocking solution of 5% normal goat serum and PBST for one hour at room temperature. The larval fillet was incubated with the primary antibody overnight at 4°C, washed 3 times with PBT, incubated with the secondary antibody for 4 hours at room temperature and then washed again 3 times with PBT. The larval fillet was mounted on a slide with glycerol to prepare for viewing. The primary antibodies used were anti-twinstar/cofilin (1∶50; gift from J.R. Bamburg), anti-actin (1∶200, A4700; Sigma, St Louis, MO), and anti-GFP (1∶1000; Molecular Probes, Euegene, OR). Staining actin with phalloidin (0.16 µM, Alexa Fluor 546 phalloidin; Invitrogen, Carlsbad, CA) was performed according to manufacturer's instructions, and when double labeling, was done after washing off the secondary antibodies.

### Image acquisition and processing

For live and fixed larva visualization of the PNS, images were obtained using a Zeiss LSM (Laser Scanning Microscope) 510 (Germany) confocal microscope with a 40× and a 63× oil immersion lens. For live specimens, larvae were picked from food bottles and vials, cleaned with water, mounted on a slide with halocarbon and covered with a cover slip. Fixed specimens were washed with 1× PBT and mounted on a slide with glycerol and covered with a cover slip. Database microscopic images taken using the LSM510 software exported as TIFF files, cropped and sized using Adobe Photoshop (San Jose, CA) and arranged and labeled using Adobe Illustrator (San Jose, CA).
